# Dentition

**Published:** 1890-03-15

**Authors:** 


### DENTITION.

The relation of dentition to diseases of the alimentary tract is the title of a paper, "Archives of Pediatrics," by Dr. Samuel S. Adams, of Washington. A series of tables are given, showing the mode of feeding of the children, and the manner and period of the eruption of the teeth. From such data the writer comes to the conclusion that teething is not a cause of the multitude of diseases from which teething children suffer. "I would not," Dr. Adams writes, "be dogmatic in the assertion that dentition is never a cause of disease, but such indeed has been my experience in pediatrics." If the investigation of the subject is pursued without that bias which the teaching of the schools inclines us to, the conclusion that improper alimentation and not teething is the factor in causing disease of the alimentary canal must be arrived at. Doubtless this is correct with regard to perhaps the great majority of infants, but on the other hand, there is no doubt that the irritation caused by teething does sometimes induce intestinal maladies, and also others more specially pertaining to the nervous system.

